# A redesigned CRISPR/Cas9 system for marker-free genome editing in *Plasmodium falciparum*

**DOI:** 10.1186/s13071-016-1487-4

**Published:** 2016-04-11

**Authors:** Junnan Lu, Ying Tong, Jiaqiang Pan, Yijun Yang, Quan Liu, Xuefang Tan, Siting Zhao, Li Qin, Xiaoping Chen

**Affiliations:** Laboratory of Pathogen Biology, State Key Laboratory of Respiratory Disease, Center for Infection and Immunity, Guangzhou Institutes of Biomedicine and Health (GIBH), Chinese Academy of Sciences, No. 190 Kaiyuan Avenue, Guangzhou Science Park, Guangzhou, 510530 Guangdong Province China; CAS Lamvac Biotech Co., Ltd, No. 3 Lanyue Road, Guangzhou Science Park, Guangzhou, 510530 Guangdong Province China

**Keywords:** Marker-free, CRISPR/Cas9, *Plasmodium falciparum*

## Abstract

**Background:**

A highly efficient CRISPR/Cas9-based marker-free genome editing system has been established in *Plasmodium falciparum* (*Pf*). However, with the current methods, two drug-selectable markers are needed for episome retention, which may present hurdles for consecutive genome manipulations due to the limited number of available selectable markers. The loading capacity of donor DNA is also unsatisfactory due to the large size of the Cas9 nuclease and sgRNA co-expression system, which limits the size of knock-in DNA fragments. Because of the inefficient end joining (EJ) DNA repair mechanism of *Pf*, a suicide-rescue approach could be used to address the challenges. Cas9 nuclease and sgRNA were co-expressed from a single plasmid (suicide vector) with one selectable marker, and the donor DNA was ligated into the other plasmid (rescue vector) containing only the ampicillin-resistance gene (AmpR) and a ColEl replication origin (ori). Nonetheless, whether this approach can mediate even the regular gene editing in *Pf* remains unknown. This study aimed to demonstrate the basic gene editing function of this Cas9-mediated suicide-rescue system.

**Findings:**

The suicide and rescue vectors were constructed and co-transfected into *Pf*3D7. This system worked as expected when used to disrupt the *Pfset2* gene and to insert a green fluorescent protein-renilla luciferase (*gfp-ruc*) fusion gene cassette of 3334 base pairs (bp) into the *Pf47* locus, demonstrating that the suicide vector actually induced double-strand breaks (DSBs) and that the rescue vector functioned without maintenance via drug selection.

**Conclusions:**

The adapted marker-free CRISPR/Cas9 system with only a single episome-selectable marker performs well as the current systems for general gene editing which lays a solid foundation for further studies including consecutive gene manipulations and large gene knock-ins.

**Electronic supplementary material:**

The online version of this article (doi:10.1186/s13071-016-1487-4) contains supplementary material, which is available to authorized users.

## Findings

### Background

Malaria is still a major public health burden in many countries, and the most lethal malaria parasite is *Plasmodium falciparum* (*Pf*) [1, 2]. An understanding of the gene function of *Pf* and the performance of pathogen genetic manipulations is essential for anti-malaria drug and malaria vaccine development. The traditional approach to genome modification in *Pf* relies on single- or double-crossover recombination [3, 4] and is time-consuming and labor intensive. Moreover, the knock-in of larger gene cassettes without selectable marker integration is impractical using such strategies.

The newly developed genome-editing technology CRISPR/Cas9 has been successfully used in *Pf* and exhibits higher efficiency. Transgenic parasites can be obtained within a typical timeframe of 3–6 weeks, and both marker-integrated and marker-free systems are available [5, 6]. In the system developed by Ghorbal et al. [5], Cas9 nuclease and sgRNA are expressed under the endogenous Hsp86 and U6 promoters and form a complex with targeted DNA strands to induce double-strand breaks (DSBs) with high efficiency. This process results is considerable target gene replacement with the drug-selectable marker-cassette human dihydrofolate reductase (h*dhfr*). Site-specific mutation without the integration of a selectable marker can also be efficiently achieved by maintaining the CRISPR/Cas9 system and the donor DNA loading plasmids as episomes using two selectable markers, h*dhfr* and yeast dihydroorotate dehydrogenase (*yDHODH*). In another CRISPR/Cas9 system for *Pf* adapted by Wagner et al. [6], T7 RNA polymerase (T7 RNAP) is introduced for sgRNA transcription. As a marker-free genome-editing tool, blasticidin S deaminase (*bsd*) and neomycin phosphotransferase (*neo*) are employed to maintain the plasmids carrying the T7 RNAP transcribe system, CRISPR/Cas9 system and donor DNA. This T7 RNAP-dependent system yields 50–100 % gene knockout frequencies within the usual time frame. The successful application of this powerful tool promises to accelerate both basic and applied *Pf* research.

However, the current CRISPR/Cas9 systems may not be competent for consecutive genome manipulations in *Pf* due to the limited number of available selection markers and potential drug incompatibility. Moreover, the loading capacity for donor DNA is also unsatisfactory; indeed, current systems may not accept larger exogenous DNA fragments for knock-in. As described above, in the system developed by Wagner et al. [6]*,* T7 RNAP is used for sgRNA transcription, and two plasmids requiring two drugs that must be simultaneously administered for episome retention are used. Additionally, the loading capacity for the donor DNA fragment is limited because the donor plasmid must accept the expression cassettes of the T7 RNAP gene and the drug-selectable marker. The system constructed by Ghorbal et al. [5] is relatively compact due to the use of the endogenous U6 promoter to transcribe the sgRNA, but it also involves two plasmids with two markers and allows limited space for the donor DNA. Although marker-free editing can now be achieved, it may be difficult to perform multiple consecutive genome manipulations, and larger gene cassette knock-ins might suffer technical hurdles.

It has been reported that none of the proteins required for the canonical non-homologous end joining (C-NHEJ) pathway have been identified in *Pf* [7–9]. The efficiency of EJ DNA repair seems extremely low, although an alternative EJ pathway has been found in *Pf* [6, 9, 10]. In view of the inefficient EJ DNA repair in *Pf*, a suicide-rescue approach might be appropriate to address these challenges. A common feature of the two systems is that Cas9 nuclease and sgRNA are not co-expressed by a single plasmid perhaps because the plasmid may be too large to be manipulated and may impair the transfection efficiency. If Cas9 nuclease and the sgRNA were encoded on the same plasmid along with the drug selection marker, the site-specific breaks could be induced by just one plasmid (a suicide vector). When the suicide vector is co-transfected with an independently established donor plasmid (a rescue vector) containing the homology arms without another selectable marker, the parasites that receive both plasmids may survive and be selected under drug pressure; the other parasites would die due to the drug or inefficient end-joining DNA repair. Because plasmids lacking the selectable marker would be lost rapidly [8], recovering parasites would theoretically suffer DNA breaks and homologous recombination would occur. The suicide vector would be maintained as an episome, and the next round of genome editing could proceed with the same system but with another drug-selectable marker. Because the donor plasmid is specialized, it can support larger sequences for knock-ins into *Pf*. The basic gene editing function of this suicide-rescue approach has been proved using ZFN-mediated gene replacement without a selectable phenotype in *Pf* [11]. However, whether the Cas9-mediated suicide-rescue system can perform regular gene editing in *Pf* remains unknown. The present study aims to confirm the regular ability of the Cas9-mediated suicide-rescue system and to provide a selectable marker saving, easily operated and low-cost marker-free genome editing tool for *Pf* that may be more suitable for consecutive gene manipulations and also be competent for larger gene cassette knock-ins.

## Methods

### Plasmid constructs

pCas9-BSD-sgRNA (pCBS), the plasmid for Cas9 nuclease, *bsd* and sgRNA co-expression, was generated in two steps. pL6BSD was first constructed by replacing the h*dhfr* cassette of pL6-eGFP [5] with the *bsd* cassette from pCC4 [12] at the restriction sites Sac II and Nco I. Then, the *yfcu*-coding sequence of pL6BSD was replaced with the Cas9 nuclease open reading frame (ORF) from pUF1-Cas9 [5] using the restriction sites Xho I and Kpn I.

For both targets, pCBS-*Pfset2* and pCBS-*Pf47* were made by replacing the BtgZ I-adaptor with guide DNA sequence as previously described [5]. The oligonucleotides used for *Pfset2* and *Pf47* were P9/P10 (Additional file 1) and P11/P12*,* respectively. pGCBS-*Pfset2* was derived from *pCBS-Pfset2* via the addition of a *Thosea asigna* virus 2A peptide (GFP-2A)-coding sequence that was amplified using P31/P32 from pBSDv2.1–3 (Additional file 2) at the restriction site Xho I upstream of the Cas9 ORF.

To produce the donor plasmid pARM-*Pfsets*, the left and right homology arms were amplified using P13/P14 and P15/P16, respectively, from the genomic DNA of *Plasmodium falciparum* strain 3D7. The two arms were ligated into an intact donor DNA via overlap PCR using P13/P16, and they were then connected to the ‘Ampicillin-resistance gene-ColE1 replication origin’ (‘AmpR-ColE1 ori’) fragment, which was amplified using P17/P18 from the commercial T-vector. To detect homologous recombination (HR), a 48-bp DNA fragment was introduced between the arms using P14 and P15. The other donor plasmid, pARM-GFP/RUCki, was constructed by ligating the ‘AmpR-ColE1 ori’ fragment with the *gfp-ruc* fusion cassette flanked by the homology arms. The left arm, right arm and *Pf* elongation factor 1-alpha (*Pfef1α;* [PlasmoDB: PF13_0304]) 5’ untranslated region (UTR) were amplified using P19/P20, P21/P22, and P23/P24 from the genomic DNA of 3D7. The *gfp* ORF, *renilla luciferase* ORF and *Plasmodium berghei* dihydrofolate reductase (*Pbdhfr*; [PlasmoDB: PBANKA_0719300]) terminator (PbDT) were amplified from pLN-GFP (Additional file 2), pHBRIH and the genomic DNA of *P. berghei ANKA* using P25/P26, P27/P28, and P29/P30, respectively. All cloning reactions used the ClonExpress II One-Step Cloning Kit (Vazyme) and XL10-competent cells (Vazyme).

### Parasite culture and transfections

*Pf* strain 3D7 parasites were routinely cultured in fresh human red blood cells under 5 % O_2_ and 5 % CO_2_ in RPMI-1640 media supplemented with 5 g/L Albumax I (Thermo Fisher Scientific), 2 g/L NaHCO_3_, 25 mM HEPES, 1 mM hypoxanthine and 50 mg/L gentamicin. The transfections used ~100 μg of each plasmid and were performed by the spontaneous DNA uptake method [13]. The transfectants were obtained by selection with 5.0 μg/mL blasticidin S (Thermo Fisher Scientific).

### Whole-cell PCR analysis

Mutant parasites were verified by PCR using PrimeSTAR GXL DNA Polymerase (Takara Bio, Inc.) according to the manufacturer’s instructions. The parasites used as PCR templates were prepared as previously described [14], and cultures at 0.1–10 % parasitaemia and 2 % hematocrit were centrifuged at 350 × *g* for 3 min. The pellets were flash-frozen in liquid nitrogen and then thawed, and 1 μL of the lysed cells was quickly added to 50 μL of the PCR reaction solution on ice to yield a final concentration of 1× PrimeSTAR GXL Buffer. The primers used to detect the *Pfset2* disruption were P1/P2, P3/P4 and P1/P4. The primers used to detect the *gfp-ruc* fusion cassette knock-in were P5/P6, P7/P8 and P5/P8. The PCR products yielded by P1/P4 and P5/P8 were purified using an agarose gel extraction kit and sequenced using the same primers.

### Fluorescence-activated cell sorting (FACS) of GFP-positive parasites

The parasite cultures were centrifuged at 350 × *g* for 3 min, washed, and resuspended with phosphate-buffered saline to a concentration of 1.0 × 10^6^ cells/mL. The GFP-positive parasites were sorted with a FACSAria™ II flow cytometer and then used for whole-cell PCR analysis as described above.

### Live cell fluorescence microscopy/laser confocal microscopy

The parasite cultures were incubated for 10 min with RPMI medium containing 2 μg/mL Hoechst 33,342 (Sigma) at 37 °C and then washed twice with PBS and applied to a 3.5-cm culture dish (Nunc). Imaging was conducted immediately at room temperature using a Nikon Ti-S inverted microscope or a Zeiss 710 NLO laser confocal microscope.

### Luciferase assay

Renilla luciferase expression was confirmed using the Renilla Luciferase Assay System (Promega). The parasite samples were prepared by centrifugation of the cultures at ~5 % parasitaemia and 2 % hematocrit. Next, 15-μL pellets were either used immediately or stored at −80°C.

## Results and discussion

**Fig. 1:**
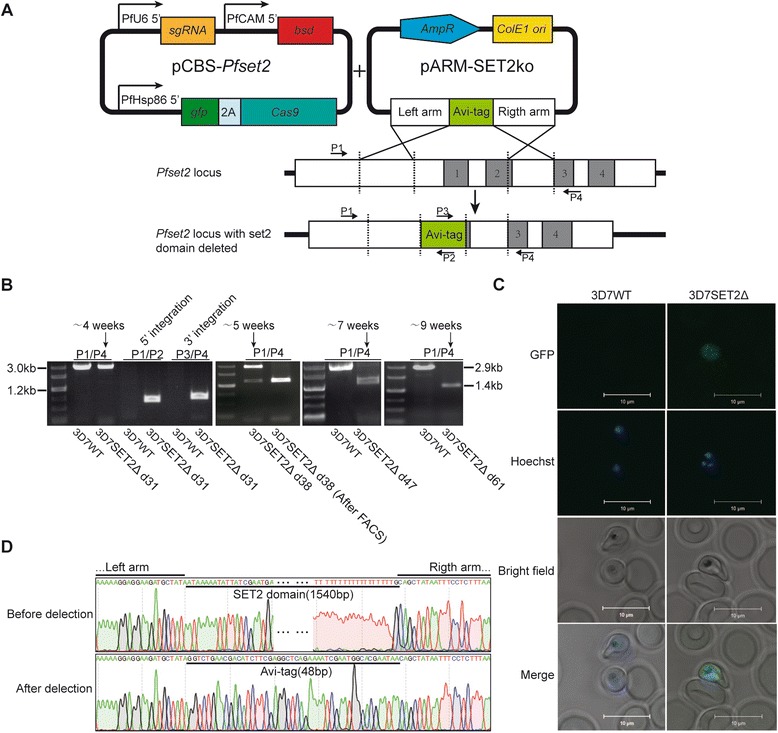
Redesigned marker-free CRISPR/Cas9-mediated deletion of the *Pfset2* locus. **a** Construct used for *Pfset2* gene disruption. Introns 1 to 4 of the *Pfset2* locus are represented as gray boxes. pGFP-CBS-*Pfset2* was designed to induce a double-strand break (DSB) near the 5’ end of intron 2. The Avi-tag between the homology arms was added to detect donor integration in the design of the PCR primers. *Pf* U6 5’, *Pf* U6 spliceosomal RNA promoter region; *Pf* CAM5’, *Pf* calmodulin promoter region; *Pf* Hsp86 5’, *Pf* heat shock protein 86 promoter region; AmpR, ampicillin resistance gene; ori, replication origin; ko, knockout. The positions and directions of the primers P1 to P4 are indicated by the small black arrows. **b** PCR analysis of the parasite populations obtained after transfection. WT, wild-type; SET2Δ, *Pfset2* knockout; d31, d38, d47, and d61, days 31, 38, 47, and 61 after transfection, respectively; FACS: fluorescence-activated cell sorting. **c** Laser confocal microscopy of the parasites expressing the GFP protein. **d** DNA sequencing confirmed a 1.5-kb deletion in the *Pfset2* gene. The top panel shows the partial nucleotide sequences of the left and right arms from the parental strain. The bottom panel shows the 48-bp DNA insert between the left and right arms

We first redesigned and successfully constructed the basic suicide vector pCas9-BSD-sgRNA (pCBS) consisting of the expression cassettes for the sgRNA, Cas9 nuclease and blasticidin S deaminase. These genes were driven by endogenous promoters. To test the function of this suicide-rescue system, *Pfset2* was chosen as a target because it has been demonstrated to be a nonessential gene for in vitro blood-stage survival of *Pf* [15]*.* The suicide vector pGFP-CBS-*Pfset2* with a *gfp* reporter gene was derived from pCBS-*Pfset2.* The donor DNA template consisting of homology arms flanking the Avi-tag was provided by a rescue plasmid, pARM-SET2ko, without a drug-selectable marker for *Pf* (Fig. 1a). pGFP-CBS-*Pfset2* and pARM-SET2ko were co-transfected, and blasticidin S selection was applied. The drug-resistant parasites were collected approximately 4 weeks after transfection, and PCR results confirmed integration of the Avi-tag. However, the truncated locus was nearly undetectable (Fig. 1b). The proportion of edited parasites exhibited an increasing upward tendency as indicated by the subsequent PCR detection (Fig. 1b). One week later, the truncated locus became highly visible (Fig. 1b), and GFP-positive parasites were observed by confocal microscopy (Fig. 1c). The GFP-positive parasites obtained by FACS were determined to be edited; no wild-type (WT) loci were detected (Fig. 1b). Additionally, sequencing results indicated the expected editing events (Fig. 1d). The PCR results at day 47 revealed that the mutant parasites became dominant, and the WT locus was nearly undetectable after day 61 (Fig. 1b). Together, these results indicate that the suicide vector actually induced DSBs and that the rescue plasmid also performed well, although it was transiently supplied. Thus, a marker-free genome editing system with only one selectable marker was successfully created. This experiment demonstrated once again that the EJ mechanism of *Pf* is very inefficient.

Currently, the following five drug-selectable markers are available for *Pf*: h*dhfr* [16]*, bsd* [17]*, neo* [17]*, yDHODH* [18], and puromycin-N-acetyltransferase (*PAC*) [19]*.* Theoretically, at least five rounds of consecutive manipulations could be supported by the suicide-rescue system with each requiring one drug for selection and without consideration for the drug compatibility. Previous systems [5, 6] might support up to 4 rounds of editing, but the Cas9 nuclease-expressing episome had to be stably maintained beyond the first round of transfection, which might lead to exogenous plasmid integration into the parasite genome.

**Fig. 2:**
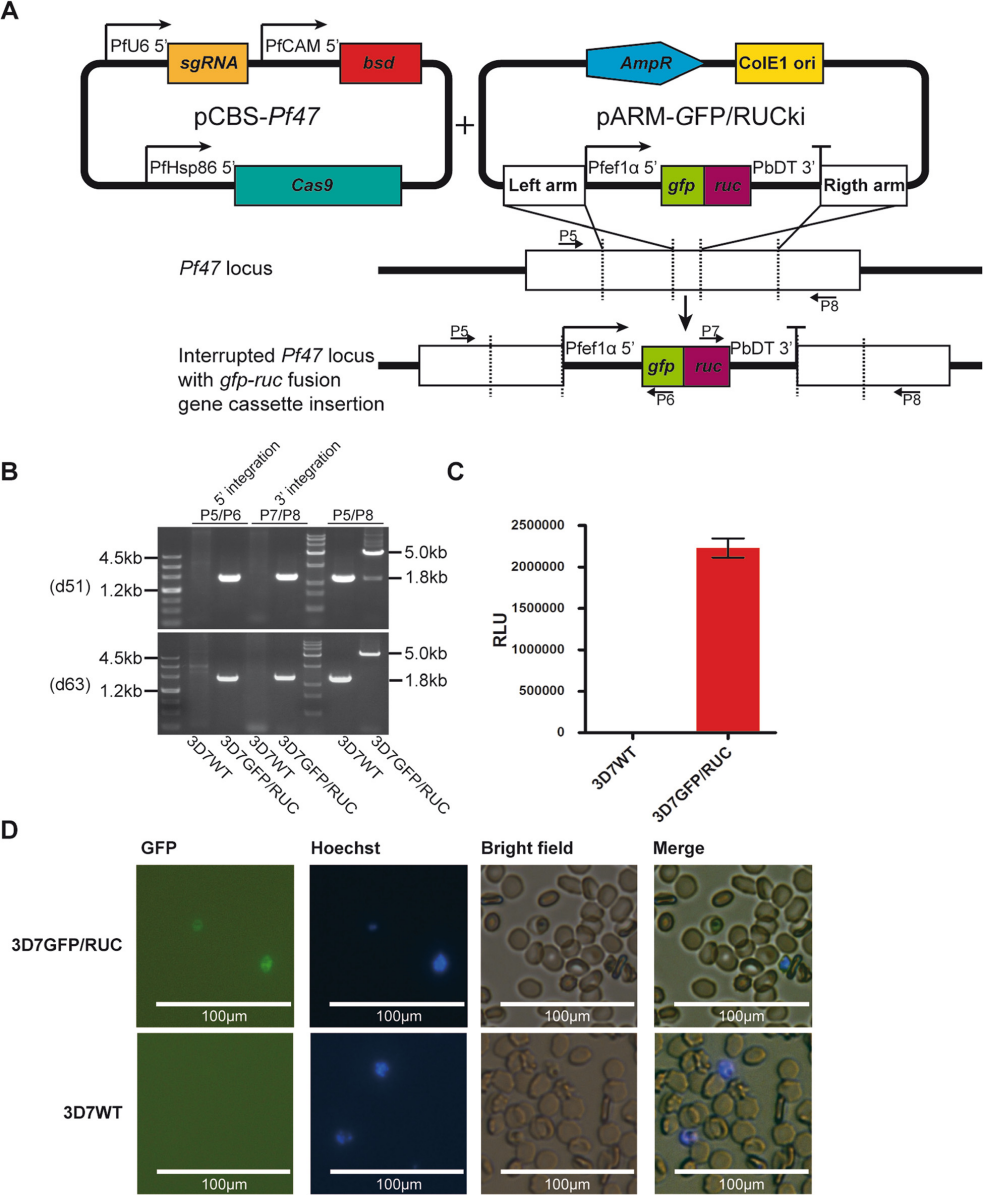
Redesigned marker-free CRISPR/Cas9-mediated insertion of the *gfp-ruc* fusion gene cassette at the *Pf47* locus. **a** Construct used for *gfp-ruc* fusion gene cassette insertion into the *Pf47* locus. pCBS-*Pf47* was designed to target the *Pf47* ORF at its 5’ terminal-region, ~150 bp from the start codon. pARM-GFP/RUCki provides a donor fragment with the *gfp-ruc* fusion cassette flanked by homology arms. The positions and directions of primers P5 to P8 are indicated by small black arrows. *Pf* EF1α 5’, *Pf* elongation factor 1-alpha promoter region; *Pb*DT 3’, *Pb* dihydrofolate reductase terminator; ki, knock-in. (**b**) PCR analysis of the parasite populations obtained after transfection. d51 and d63, days 51 and 63 after transfection, respectively. (**c**) Luciferase assay confirming the functional expression of the integrated *ruc* gene in the parasites obtained after transfection. The negative control was 3D7 WT. The luciferase assays were performed in triplicate, and the standard deviations are indicated by vertical bars. (**d**) Live cell fluorescence microscopy of the parasites expressing the GFP protein. The negative control was 3D7 WT

The suicide-rescue editing system has also been used to mediate the insertion of a *gfp-ruc* fusion gene cassette (a 3337-bp DNA fragment) into the *Pf47* locus of *Pf* [20] (Fig. 2a). Live parasites were obtained approximately 4 weeks after transfection. Luciferase assay and fluorescence microscopy results indicated that both genes were functionally expressed (Fig. 2c, d). PCR and sequencing confirmed the insertion of the fusion gene cassette. The WT locus was still detectable at day 51 but was undetectable approximately 12 days later (Fig. 2b). A parallel transfection experiment yielded similar results, and the WT locus was also undetectable by PCR at day 60. These results illustrate that the redesigned system was capable of mediating the knock-in of larger exogenous gene cassettes and that the extended size of the donor plasmid did not obviously impair the efficiency of co-transfection. Exogenous gene knock-in and endogenous add-back are typically performed in studies of *Pf*, but the donor DNA loading capacities of previous systems might limit the sizes of inserted fragments to 10 kilobases (kb) for the donor DNA loading plasmids have exceeded 9.0 kb [5] or even 11.0 kb [6]. The rescue plasmid used in this study contains only a 2.1-kb ‘AmpR-ori’ backbone and thus is capable of providing 7.0 kb or 9 kb of extra capacity.

The editing efficiency was not directly assayed in this study. Obviously, the proportion of the parasite population that exhibited editing was dependent on the drug selection time as was observed in both the knock-out and knock-in experiments. According to the previous report, high gene-disruption frequencies (≥ 50–100 %) can be achieved within the usual timeframe (4–6 weeks) using marker-free CRISPR/Cas9 editing [6]. In our study, the edited population became dominant (≥ 50 %) in approximately 6–7 weeks, as demonstrated by PCR analysis, and this timeframe is similar to the typical timeframe. Thus it can be seen that the regular drug selection time is not always sufficient for obtaining highly pure edited population (~100 % editing efficiency) in both previous and redesigned system. In order to avoid the possible plasmid integration, limit dilution and culture without drug pressure should be employed when the gene modified parasites are detectable within the usual timeframe. This process usual needs nearly a month of time for obtaining single-clone parasites with considerable amount for genetic analysis [21] and for next round gene editing. However, this strategy is time consuming and labor intensive because multi-round limited dilution would be needed in consecutive gene manipulation. An alternative method is a compact gene editing process without interruption with limited dilution following the last round manipulation, but the prerequisite is that the highly pure edited population has been generated in the previous gene manipulation. Thus in our study the drug selection time was extended to confirm whether the highly pure edited population could be obtained and how long it would take. In both experiments the time required for the total elimination of the WT parasites (i.e. below the level detectable by PCR) was approximately 9 weeks. This strategy shows no advantages in time consumption compared with limited dilution dependent approach, unless the selection time could be reduced to the usual timeframe. It can be speculated that this extended elimination time was attributable to spontaneous drug resistance in the WT parasite. The GFP-positive parasites obtained by FACS in the knock-out test were part of a highly pure mutant population, which indicated that the 2.9-kb untruncated locus was amplified from spontaneously occurring blasticidin-resistant parasites. It has been reported that blasticidin pressure results in resistant transport mutant selection in the FCB strain of *Pf* [22], and 3D7 may also acquire blasticidin resistance through mutation. The time required to reach 100 % edited parasites might be reduced by appropriately increasing the blasticidin concentration.

In conclusion, a suicide-rescue-based marker-free CRISPR/Cas9 system was developed and confirmed to be competent for general gene manipulations in *Pf*. This system requires fewer selectable markers and exhibits potential for large gene cassette knock-ins. This tool may be a useful alternative to *Pf* genome editing. However, further experiments are needed to confirm whether this system could efficiently mediate consecutive gene manipulations and large gene cassette knock-ins. Although a CRISPR/Cas9 system has been established in *Pf*, we call for additional studies to increase the efficiency and versatility of this system.

## Additional files

Additional file 1: PCR primers used in this study. (PDF 35 kb) Additional file 2: sequences of pBSDv2.1-3 and pLN-GFP. (PDF 60 kb)

## Abbreviations

2A: *Thosea asigna* virus 2A peptide
*bsd*: blasticidin S deaminase
C-NHEJ: canonical non-homologous end joining
DSBs: double-strand breaks
EJ: end joining
FACS: fluorescence-activated cell sorting
*gfp-ruc*: green fluorescent protein-renilla luciferase
h*dhfr*: human dihydrofolate reductase
HR: homologous recombination
*neo*: neomycin phosphotransferase
*PAC*: puromycin-N-acetyltransferase
*Pb*: *Plasmodium berghei*
*Pf*: *Plasmodium falciparum*
T7 RNAP: T7 RNA polymerase
*yDHODH*: yeast dihydroorotate dehydrogenase
y*fcu*: *y*east cytosine deaminase and uridyl phosphoribosyl transferase
