# Erratum to: A redesigned CRISPR/Cas9 system for marker-free genome editing in *Plasmodium falciparum*

**DOI:** 10.1186/s13071-016-1580-8

**Published:** 2016-05-17

**Authors:** Junnan Lu, Ying Tong, Jiaqiang Pan, Yijun Yang, Quan Liu, Xuefang Tan, Siting Zhao, Li Qin, Xiaoping Chen

**Affiliations:** Laboratory of Pathogen Biology, State Key Laboratory of Respiratory Disease, Center for Infection and Immunity, Guangzhou Institutes of Biomedicine and Health (GIBH), Chinese Academy of Sciences, No. 190 Kaiyuan Avenue, Guangzhou Science Park, Guangzhou, 510530 Guangdong Province China; CAS Lamvac Biotech Co., Ltd., No. 3 Lanyue Road, Guangzhou Science Park, Guangzhou, 510530 Guangdong Province China

In our recently published article [1], we noticed incorrect citations, typing errors and improper punctuations. Thus, “pCBS-*Pfset2*” and “pGFP-CBS-*Pfset2*” were both used in our original article, but in Fig. 1a the name “pCBS-*Pfset2*” was wrongly cited; this should read “pGFP-CBS-*Pfset2*”. The correct version of Fig. 1 can be found below. In other sections, “pCBS-*Pfset2*” was cited correctly. In the “Plasmid constructs” section of the original article, “pGCBS-*Pfset2*” should read “pGFP-CBS-*Pfset2*”, and “pARM-*Pfset2*”, the name of the rescue plasmid for *Pfset2* gene disruption, should read “pARM-SET2ko”. We would like to apologize for the errors and for any inconvenience this may have caused.

**Fig. 1:**
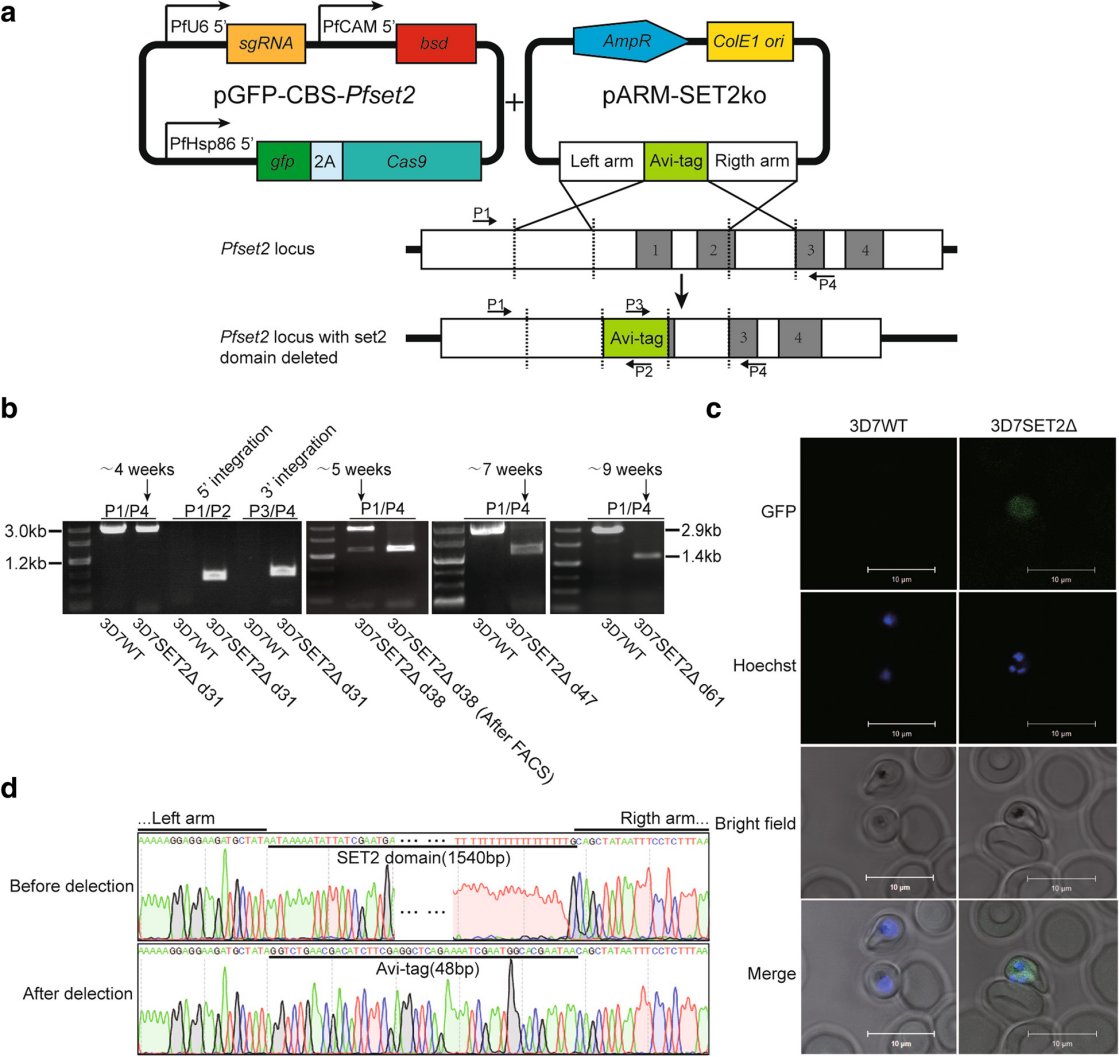
Redesigned marker-free CRISPR/Cas9-mediated deletion of the *Pfset2* locus. **a** Construct used for *Pfset2* gene disruption. Introns 1 to 4 of the *Pfset2* locus are represented as gray boxes. pGFP-CBS-*Pfset2* was designed to induce a double-strand break (DSB) near the 5' end of intron 2. The Avi-tag between the homology arms was added to detect donor integration in the design of the PCR primers. *Pf* U6 5’, *Pf* U6 spliceosomal RNA promoter region; *Pf* CAM5’, *Pf* calmodulin promoter region; *Pf* Hsp86 5’, *Pf* heat shock protein 86 promoter region; AmpR, ampicillin resistance gene; ori, replication origin; ko, knockout. The positions and directions of the primers P1 to P4 are indicated by the small black arrows. **b** PCR analysis of the parasite populations obtained after transfection. WT, wild-type; SET2Δ, *Pfset2* knockout; d31, d38, d47, and d61, days 31, 38, 47, and 61 after transfection, respectively; FACS: fluorescence-activated cell sorting. **c** Laser confocal microscopy of the parasites expressing the GFP protein. **d** DNA sequencing confirmed a 1.5-kb deletion in the *Pfset2* gene. The top panel shows the partial nucleotide sequences of the left and right arms from the parental strain. The bottom panel shows the 48-bp DNA insert between the left and right arms
