# Supplementary material for: A redesigned CRISPR/Cas9 system for marker-free genome editing in Plasmodium falciparum
Source: Parasit Vectors. 2016 Apr 11;9:198. doi: 10.1186/s13071-016-1487-4 (PMC4828878; doi:10.1186/s13071-016-1487-4)
Supplement: Additional file 1: — PCR primers used in this study. (PDF 35 kb) [file 13071_2016_1487_MOESM1_ESM.pdf]

Additional file 1: PCR primers used in this study

primer name	sequence (5’ to 3’)
P1	CACTATTATTTAGGAGCTTCAGCATATTGTGTACCTTTAGACAGG
P2	CCATTCGATTTTCTGAGCCTCGAAGATG
P3	CATCTTCGAGGCTCAGAAAATCGAATGG
P4	CCTTTCACATTTATACTCAAGCTCCAATCCAATTATGTTATGCAGG
P5	CCATTTCTTGCTTCAGTTATGTAGATACTATGATG
P6	CATCACCTTCACCCTCTCCAC
P7	AATGCTTATCTACGTGCAAGTGATG
P8	TGGTATTAATTTATATGGACATTTAATGCCTGC
P9	gctatttctagctctaaaacATGGAATATCTCTCAAGGCA
P10	catattaagtatataatattTGCCTTGAGAGATATTCCAT
P11	catattaagtatataatattATGTTAAGCCAACTGTAGTT
P12	gctatttctagctctaaaacAACTACAGTTGGCTTAACAT
P13	GCAGATCTAAGCTTCTCGAGATAAGGAAAATCAAATTGAACCAGTAAACGG
P14	CGATTTTCTGAGCCTCGAAGATGTCGTTCAGACCTATAGCATCTTCCTCCTTTTTAC
P15	CTTCGAGGCTCAGAAAATCGAATGGCACGAATAACAGCTATAATTTCCTCTTTAATAAC
P16	GCGGCCGCGGTACCGGATCCCTGTTGTATTCTTCTCAGCTGACC
P17	CTCGAGAAGCTTAGATCTGCTCTTCCGCTTCCTCGCTCAC
P18	GGATCCGGTACCGCGGCCGCTCCGGGAGCTGCATGTGTCAG
P19	GCAGATCTAAGCTTCTCGAGGAGCCGTATGATTTATCATTTG
P20	AAGAGGGGTGAACCGTTAAACACATACGTATTGTGTTGAG
P21	ACTTTTCTTCTTCAGGGTAGGGATGTACTATAAACAACCCTAC
P22	GCGGCCGCGGTACCGGATCCGATCTTGAGATGAAAGCATAC
P23	TTTAACGGTTCACCCCTCTTAAC
P24	TTTGAAATAATTTTAATTTTTTTTTTTTTTAAATAG
P25	AAAATTAAAATTATTTCAAAATGAGTAAAGGAGAAGAACTTTTC
P26	GTCATGCCGGACGGACCACCTTTGTATAGTTCATCCATGCC
P27	GGTGGTCCGTCCGGCATGACTTCGAAAGTTTATGATC
P28	ATAAATAAGAAAAACGAACATTATTGTTCATTTTTGAGAACTCG
P29	TGTTCGTTTTTCTTATTTATATATTTATACC
P30	CTACCCTGAAGAAGAAAAGTC
P31	CACATTTCGAATAAACTCGAGATGAGTAAAGGAGAAGAACTTTTCAC
P32	GTCCTTATAGTCCATCTCGAGCGATTCTGGGCCAGGATTCTCCTC
