# Supplementary material for: A redesigned CRISPR/Cas9 system for marker-free genome editing in Plasmodium falciparum
Source: Parasit Vectors. 2016 Apr 11;9:198. doi: 10.1186/s13071-016-1487-4 (PMC4828878; doi:10.1186/s13071-016-1487-4)
Supplement: Additional file 2: — sequences of pBSDv2.1-3 and pLN-GFP. (PDF 60 kb) [file 13071_2016_1487_MOESM2_ESM.pdf]

Additional file 2: sequences of pBSDv2.1-3 and pLN-GFP

sequence of pBSDv2.1-3

GAGTATTCTATAGTGTCACCTAAATAGCTTGGCGTAATCATGGTCATAGCTGT
TTCCTGTGTGAAATTGTTATCCGCTCACAATTCCACACAACATACGAGCCGG
AAGCATAAAGTGTAAAGCCTGGGGTGCCTAATGAGTGAGCTAACTCACATT
AATTGCGTTGCGCTCACTGCCCGCTTTCCAGTCGGGAAACCTGTCGTGCCA
GCTGCATTAATGAATCGGCCAACGCGCGGGGAGAGGCGGTTTGCGTATTGG
GCGCTCTTCCGCTTCCTCGCTCACTGACTCGCTGCGCTCGGTCGTTCGGCT
GCGGCGAGCGGTATCAGCTCACTCAAAGGCGGTAATACGGTTATCCACAGA
ATCAGGGGATAACGCAGGAAAGAACATGTGAGCAAAAGGCCAGCAAAAG
GCCAGGAACCGTAAAAAGGCCGCGTTGCTGGCGTTTTTCGATAGGCTCCGC
CCCCCTGACGAGCATCACAAAAATCGACGCTCAAGTCAGAGGTGGCGAAA
CCCGACAGGACTATAAAGATACCAGGCGTTTCCCCCTGGAAGCTCCCTCGT
GCGCTCTCCTGTTCCGACCCTGCCGCTTACCGGATACCTGTCCGCCTTTCTC
CCTTCGGGAAGCGTGGCGCTTTCTCATAGCTCACGCTGTAGGTATCTCAGTT
CGGTGTAGGTCGTTCGCTCCAAGCTGGGCTGTGTGCACGAACCCCCCGTTC
AGCCCGACCGCTGCGCCTTATCCGGTAACTATCGTCTTGAGTCCAACCCGG
TAAGACACGACTTATCGCCACTGGCAGCAGCCACTGGTAACAGGATTAGCA
GAGCGAGGTATGTAGGCGGTGCTACAGAGTTCTTGAAGTGGTGGCCTAACT
ACGGCTACACTAGAAGGACAGTATTTGGTATCTGCGCTCTGCTGAAGCCAG
TTACCTTCGGAAAAAGAGTTGGTAGCTCTTGATCCGGCAAACAAACCACC
GCTGGTAGCGGTGGTTTTTTTGTTTGCAAGCAGCAGATTACGCGCAGAAAA
AAAGGATCTCAAGAAGATCCTTTGATCTTTTCTACGGGGTCTGACGCTCAG
TGGAACGAAAACTCACGTTAAGGGATTTTGGTCATGAGATTATCAAAAAGG
ATCTTCACCTAGATCCTTTTAAATTAAAAATGAAGTTTTAAATCAATCTAAA
GTATATATGAGTAAACTTGGTCTGACAGTTACCAATGCTTAATCAGTGAGGC
ACCTATCTCAGCGATCTGTCTATTTCGTTCATCCATAGTTGCCTGACTCCCCG
TCGTGTAGATAACTACGATACGGGAGGGCTTACCATCTGGCCCCAGTGCTG
CAATGATACCGCGAGACCCACGCTCACCGGCTCCAGATTTATCAGCAATAA
ACCAGCCAGCCGGAAGGGCCGAGCGCAGAAGTGGTCCTGCAACTTTATCC
GCCTCCATCCAGTCTATTAATTGTTGCCGGGAAGCTAGAGTAAGTAGTTCGC
CAGTTAATAGTTTGCGCAACGTTGTTGGCATTGCTACAGGCATCGTGGTGTC
ACGCTCGTCGTTTGGTATGGCTTCATTCAGCTCCGGTTCCCAACGATCAAG
GCGAGTTACATGATCCCCCATGTTGTGCAAAAAAGCGGTTAGCTCCTTCGG
TCCTCCGATCGTTGTCAGAAGTAAGTTGGCCGCAGTGTTATCACTCATGGTT
ATGGCAGCACTGCATAATTCTCTTACTGTCATGCCATCCGTAAGATGCTTTTC
TGTGACTGGTGAGTACTCAACCAAGTCATTCTGAGAATAGTGTATGCGGCG
ACCGAGTTGCTCTTGCCCGGCGTCAATACGGGATAATACCGCGCCACATAG
CAGAACTTTAAAAGTGCTCATCATTGGAAAACGTTCTTCGGGGCGAAAACT
CTCAAGGATCTTACCGCTGTTGAGATCCAGTTCGATGTAACCCACTCGTGC
ACCCAACTGATCTTCAGCATCTTTTACTTTCACCAGCGTTTCTGGGTGAGCA
AAAACAGGAAGGCAAAATGCCGCAAAAAAGGGAATAAGGGCGACACGGA
AATGTTGAATACTCATACTCTTCCTTTTTCAATATTATTGAAGCATTTATCAG

GGTTATTGTCTCATGAGCGGATACATATTTGAATGTATTTAGAAAAATAAAC
AAATAGGGGTTCCGCGCACATTTCCCCGAAAAGTGCCACCTGACGTCTAAG
AAACCATTATTATCATGACATTAACCTATAAAAATAGGCGTATCACGAGGCC
CTTTCGTCTCGCGCGTTTCGGTGATGACGGTGAAAACCTCTGACACATGCA
GCTCCCGGAGACGGTCACAGCTTGTCTGTAAGCGGATGCCGGGAGCAGAC
AAGCCCGTCAGGGCGCGTCAGCGGGTGTTGGCGGGTGTCGGGGCTGGCTT
AACTATGCGGCATCAGAGCAGATTGTACTGAGAGTGCACCATATGCTATTTC
ATCTATTTATGGACAATGGATTTATTATTATATCACACATAATATATTAAATGAT
ATAAAATATATATATATATATATATATATATATATATATATATATATATTATATTAATCAT
AATAATTATAGTATTTAATAACATTATAATTTTTATTATTTTTATTTTTTTTTTTT
TTTCAAAAGATAGATAATTTTAAAAATTATTATATATGAATTACAAATATTGCA
TAAAGATTATTATAGTATTATTATATTTATTATAATAAAATAATATATGTATGTAC
ACAATTATTTATAAGTCCAAAAATAATATATTTTACAAAAAAAAAAAAAAAAT
ATTAAAAAACAAAAACATTAATATATATATTATATAATATAATATATTTATTTATA
TATATATGTATTTTTTTTTTTATGGTAGCCTTAAAAACTTCATTATATTTAAAAT
ATATATATATATATATATATATATATATATATATTATTTTATAGGAAATAATAAAAA
AGCACCGACTCGGTGCCACTTTTTCAAGTTGATAACGGACTAGCCTTATTTT
AACTTGCTATTTCTAGCTCTAAAACTGTCGGGCAGCATCGCGAGCGATGAG
TTCCTAGGAATATTATATACTTAATATGAAATATGTGCATATAGGAAAAATTAT
GCATTTTGGTTACTCTAATATTATATATATATATATATATATATATATATATTATAATAT
ATTATGTTATATATACATAACATATACATTTTTTAATAATAATTTACCCTTTATTT
TTACATTATAAAAAATTATATTACAGTAAAAATAAAAGTTTATTATATTAATAG
TTTTTTTTTTTTTTTTTTTAATTTATGAAATATTTAAATATTTAAAATTTTTTAA
ATGAATAATTATATTTATAATTAGAAAAAAAAAAAAAAAAAAAAAAAAAAAA
ATATAGCTATTTATATAAATTTCTTTTATTTATCTGAACAAGCAAGAATTTTTT
TTTATATTAAATTAGAATAAATTATTATTAGTTTATGTATATATTTTTTTTTTTTC
ATAGTATATAAATATTATATATATTGTACCTTTTTACAATATATTTCATATATAGA
AGAGAAAAAAAAAAAAAAGAAGATATTATTGTAAAACCTCAAGATGTGTAG
AAATCCAAATGTCATGAAATTGTTCTATTTATATAATAGTTCCTTTTAATACAC
ACAAAAAAAAAAAACATGTATGTATATAATAACTACCAATTTAATAATTTGCAT
CCATAAATAATTGTAGTACACATACTATGTGACATAATAACAAAATATTACTT
TATCTTCGTTTATTTTATTATTTCGTCTTTTTTTTTTTTTTTTTTATTTATAAAAT
ATGTTTAAAATTTATAACGTCTTTTAAAGTTTAATTAAAGGAAAAAAAAAAA
AAAAAAAAAAAAAAAAAAAGAAATAAAGAAAGAAATAAAGAAATAAAGGA
ATTATATAAAAAAATACAAAAATTAAATAAAAAGATGCAATAATAAATAAAT
AGCATTAAAAAAAAAAAAATAATAAAAAATATACATACTTATTATATAAGTATA
ATTACATATGTATATTTATTTTATTTCCTTTTTAATTATTACTTTTTTATTGGTTT
TTGGCTTACATACATTAGAATAAAAACAAAATAATAACATTTGCTTTCTTGA
AACGGTTGTCCCTTTGATAATATATATATATATATACATATGTGAAAAATTATT
GTGATAATTCGTGTTATTATTTTTACCGTTCCATGGAGTTATCGATATGAATTC
TAGATTTAATAAATATGTTCTTATATATAATGAGAAATAAATATTTAACATATG
TTTAAAAAGAAAAATTTAAGATTTACATGATTAGGAATAAAAATATTAAAAG
CTATATATTAATGTTGTACTTTATGTTCATATTGTAAAAGATAAATATAATTTTT
ATTAAAATTTATATTTATTATTATTATAAGTTCTTAGAAAATACATTTTGTTTTA

AAATAATACTATAAAAAGAACAATAATAAAAATAAAATACAATTAAAACATA
ATTAAAAAAGAAAAATACAGACAGTAAAAAAAATCGCTATCCCATAAATTA
CAAAACATGAATTAATAAACATATATTGTTTATTAATAAAATTGTAAGTTATTT
TTATATATACATAATAAATTAAAGATACTGAAACAATAATTTTAAATGTAAATA
AATGAATAATGATATGTTTTATATGATTCATTATTCTATATTTATAAGGAAGATT
ACAAAAAAAAAAATTCATATGTATTTTTTTTGTAATTTCTGTGTTTATGTTTTTA
TTTTTATAATATTTTTAATCTATTATTAAATAAGCTTAGCCCTCCCACACATAA
CCAGAGGGCAGCAATTCACGAATCCCAACTGCCGTCGGCTGTCCATCACTG
TCCTTCACTATCGCTTTGATCCCAGGATGCAGATCGAGAAGCACCTGTCGG
CACCGTCCGCAGGGGCTCAAGATGCCCCTGTTCTCATTTCCGATCGCGACG
ATACAAGTCAGGTTGCCAGCTGCCGCAGCAGCAGCAGTGCCCAGCACCAC
GAGTTCTGCACAAGGTCCCCCAGTAAAATGATATACATTGACACCAGTGAA
GATGCGGCCGTCGCTAGAGAGAGCTGCGCTGGCGACGCTGTAGTCTTCAG
AGATGGGGATGCTGTTGATTGTAGCCGTTGCTCTTTCAATGAGGGTGGATTC
TTCTTGAGACAAAGGCTTATGCATAAAAAGGATCCTGATATATTTCTATTAG
GTATTTATTATTATAAAATATAAATCTTGAATGATAATAAATAAAATATTAGTTA
TTCCTTTTCTAGTTTAAAATATACATATTATAAATATATATATATATATATATATTT
TTATTGTGACAAGAATATATAATTATAAATTATATTATTTATTTTTGTATTTTTT
TTTTTTTTTTTTTTTTTTTCTTTTTTTGTTTTATTTTTCTTTTTTTTTTATAAATAT
TATTTTTTTCTTTTATCATGCACATTGGAATAATACATTAATATATATATATATAT
TATATTATACATATATTGAATAATGTTTATAAAAAATGCATAACTTATATGAATA
TAATTTTTTTTAAATATGACAAAAAGAAAAAAAAAAAAAAACCAAAAAAAA
TTAAAATTGAAATGAAATATATAAATATATTATTTATATATATTATACATTGTTT
AATACTACTACATGTATATATATATATTATATATATATATATATATCAATTTTTTCA
AAAATAAATTAATATAAAAAGAGGGAAAAAAAAAAAAAAAAAAAAAA
AAGATAATTAAGTAAGCATTTAAAAATATATAAATTGATAATATATAAAATTAA
TCACATATAAACTAATATAATTTATAAAATAAGGAAAATAAAATATTACCATA
AAATAAAAATAAAAATAAAAAAAAAAAAAAAAAAACACCTTTTTTTATATATA
TTAATATATAATTATCTCTTAGAAAAAATATTGTATAATTATATATGTAATGATT
TATATAAAAAAATAAAATTATACAAGTATATATTTTGTTTCTATAAATTGATAT
CTTAATTATTTATTATTAGAAATAGATATTTTTATAATAAACCAATAGATAAAA
TTTGTAGAGAAAAAAAAATAAAAATAAAAATAAAAATAATATAATATATAAT
AAAATAAAATAATATTATATAAATATATTTTAATTTTTTTTACAAAATGCTTAA
GACAGATCTTCGGACTAGTCCTCCCCGCGGAAAGGGGCCATTGGATATATAT
TTAGTATTCCATAGTATATAGTTAAATATAATAATATTATAGTAAATTTTTGCAT
GAATTTAAAGAAAAAAAAAAAAAAAAATAACAAAAAGGAGTATTATATAAAT
ATAACAAAATAATAAATACTATACCTATATATATATATATATATATATACATATAT
ATAAATATATAAATATATTTATATAAAAATAATATTTTATATATAATATTATATTTT
TATAATATCTTTTTTTTTTTAAATATCATCTTTCTTTCTTTCTTTCTTTTTTTTTT
TTTTTGATTTATATATAATTTTATTTTGTATTTACAAAATATAATAAAATAATTT
CATGTTTAGCAAAAATTTTAAATAAAAAATAATAATATAAAATAAAAAGCAT
CATTTAATATAAAATAATAAAATTAAAATAAAATAAGATAATTACCAAAAATA
ATAAAAAAAATTAAAAAGTATTAAAAAATATATATCATATATAAATATATAAAT
TTTAACAACGTGAGAAATTAAGAAAAAAAATTATATTTATATAAATAATATAAT

ATTTAATATATATCTCATATAATATTATATATATATATATATATATATATATTTTTTAAA
AAAAAAGAAAAAATAAGTTTATTTTTACCTTTTAATAAAAACATATTTTTAG
ATATACATAAAAATTTTTATATTATTTTATTGGTATATATATATATATATATATATA
TATAATAATATATATATATAATAAAATATATAAATATTTACATAAATATTTTACTTTA
AAAAGAAATTTTCCTTTTTTATTATATTTTTTTTCTTCCCACATTTCGAATAAA
CTCGAGATGAGTAAAGGAGAAGAACTTTTCACTGGAGTTGTCCCAATTCTT
GTTGAATTAGATGGTGATGTTAATGGGCACAAATTTTCTGTCAGTGGAGAG
GGTGAAGGTGATGCAACATACGGAAAACTTACCCTTAAATTTATTTGCACTA
CTGGAAAACTACCTGTTCCATGGCCAACACTTGTCACTACTTTCGGTTATGG
TGTTCAATGCTTTGCGAGATACCCAGATCATATGAAACAGCATGACTTTTTC
AAGAGTGCCATGCCCGAAGGTTATGTACAGGAAAGAACTATATTTTTCAAA
GATGACGGGAACTACAAGACACGTGCTGAAGTCAAGTTTGAAGGTGATAC
CCTTGTTAATAGAATCGAGTTAAAAGGTATTGATTTTAAAGAAGATGGAAAC
ATTCTTGGACACAAATTGGAATACAACTATAACTCACACAATGTATACATCA
TGGCAGACAAACAAAAGAATGGAATCAAAGTTAACTTCAAAATTAGACAC
AACATTGAAGATGGAAGCGTTCAACTAGCAGACCATTATCAACAAAATACT
CCAATTGGCGATGGCCCTGTCCTTTTACCAGACAACCATTACCTGTCCACAC
AATCTGCCCTTTCGAAAGATCCCAACGAAAAGAGAGACCACATGGTCCTTC
TTGAGTTTGTAACAGCTGCTGGGATTACACATGGCATGGATGAACTATACAA
AGGTTCGGGAGAGGGCAGAGGATCCCTGCTAACATGCGGTGATGTCGAGG
AGAATCCTGGCCCAGAATCGCTCGAGATGGACTATAAGGACCACGACGGA
GACTACAAGGATCATGATATTGATTACAAAGACGATGACGATAAGATGGCC
CCAAAGAAGAAGCGGAAGGTCGGTATCCACGGAGTCCCAGCAGCCGACA
AGAAGTACAGCATCGGCCTGGACATCGGCACCAACTCTGTGGGCTGGGCC
GTGATCACCGACGAGTACAAGGTGCCCAGCAAGAAATTCAAGGTGCTGGG
CAACACCGACCGGCACAGCATCAAGAAGAACCTGATCGGAGCCCTGCTGT
TCGACAGCGGCGAAACAGCCGAGGCCACCCGGCTGAAGAGAACCGCCAG
AAGAAGATACACCAGACGGAAGAACCGGATCTGCTATCTGCAAGAGATCT
TCAGCAACGAGATGGCCAAGGTGGACGACAGCTTCTTCCACAGACTGGAA
GAGTCCTTCCTGGTGGAAGAGGATAAGAAGCACGAGCGGCACCCCATCTT
CGGCAACATCGTGGACGAGGTGGCCTACCACGAGAAGTACCCCACCATCT
ACCACCTGAGAAAGAAACTGGTGGACAGCACCGACAAGGCCGACCTGCG
GCTGATCTATCTGGCCCTGGCCCACATGATCAAGTTCCGGGGCCACTTCCTG
ATCGAGGGCGACCTGAACCCCGACAACAGCGACGTGGACAAGCTGTTCAT
CCAGCTGGTGCAGACCTACAACCAGCTGTTCGAGGAAAACCCCATCAACG
CCAGCGGCGTGGACGCCAAGGCCATCCTGTCTGCCAGACTGAGCAAGAGC
AGACGGCTGGAAAATCTGATCGCCCAGCTGCCCGGCGAGAAGAAGAATGG
CCTGTTCGGAAACCTGATTGCCCTGAGCCTGGGCCTGACCCCCAACTTCAA
GAGCAACTTCGACCTGGCCGAGGATGCCAAACTGCAGCTGAGCAAGGACA
CCTACGACGACGACCTGGACAACCTGCTGGCCCAGATCGGCGACCAGTAC
GCCGACCTGTTTCTGGCCGCCAAGAACCTGTCCGACGCCATCCTGCTGAGC
GACATCCTGAGAGTGAACACCGAGATCACCAAGGCCCCCCTGAGCGCCTC
TATGATCAAGAGATACGACGAGCACCACCAGGACCTGACCCTGCTGAAAG
CTCTCGTGCGGCAGCAGCTGCCTGAGAAGTACAAAGAGATTTTCTTCGACC

AGAGCAAGAACGGCTACGCCGGCTACATTGACGGCGGAGCCAGCCAGGA
AGAGTTCTACAAGTTCATCAAGCCCATCCTGGAAAAGATGGACGGCACCG
AGGAACTGCTCGTGAAGCTGAACAGAGAGGACCTGCTGCGGAAGCAGCG
GACCTTCGACAACGGCAGCATCCCCCACCAGATCCACCTGGGAGAGCTGC
ACGCCATTCTGCGGCGGCAGGAAGATTTTTACCCATTCCTGAAGGACAACC
GGGAAAAGATCGAGAAGATCCTGACCTTCCGCATCCCCTACTACGTGGGCC
CTCTGGCCAGGGGAAACAGCAGATTCGCCTGGATGACCAGAAAGAGCGAG
GAAACCATCACCCCCTGGAACTTCGAGGAAGTGGTGGACAAGGGCGCTTC
CGCCCAGAGCTTCATCGAGCGGATGACCAACTTCGATAAGAACCTGCCCAA
CGAGAAGGTGCTGCCCAAGCACAGCCTGCTGTACGAGTACTTCACCGTGTA
TAACGAGCTGACCAAAGTGAAATACGTGACCGAGGGAATGAGAAAGCCCG
CCTTCCTGAGCGGCGAGCAGAAAAAGGCCATCGTGGACCTGCTGTTCAAG
ACCAACCGGAAAGTGACCGTGAAGCAGCTGAAAGAGGACTACTTCAAGA
AAATCGAGTGCTTCGACTCCGTGGAAATCTCCGGCGTGGAAGATCGGTTCA
ACGCCTCCCTGGGCACATACCACGATCTGCTGAAAATTATCAAGGACAAGG
ACTTCCTGGACAATGAGGAAAACGAGGACATTCTGGAAGATATCGTGCTGA
CCCTGACACTGTTTGAGGACAGAGAGATGATCGAGGAACGGCTGAAAACC
TATGCCCACCTGTTCGACGACAAAGTGATGAAGCAGCTGAAGCGGCGGAG
ATACACCGGCTGGGGCAGGCTGAGCCGGAAGCTGATCAACGGCATCCGGG
ACAAGCAGTCCGGCAAGACAATCCTGGATTTCCTGAAGTCCGACGGCTTC
GCCAACAGAAACTTCATGCAGCTGATCCACGACGACAGCCTGACCTTTAA
AGAGGACATCCAGAAAGCCCAGGTGTCCGGCCAGGGCGATAGCCTGCACG
AGCACATTGCCAATCTGGCCGGCAGCCCCGCCATTAAGAAGGGCATCCTGC
AGACAGTGAAGGTGGTGGACGAGCTCGTGAAAGTGATGGGCCGGCACAA
GCCCGAGAACATCGTGATCGAAATGGCCAGAGAGAACCAGACCACCCAGA
AGGGACAGAAGAACAGCCGCGAGAGAATGAAGCGGATCGAAGAGGGCAT
CAAAGAGCTGGGCAGCCAGATCCTGAAAGAACACCCCGTGGAAAACACC
CAGCTGCAGAACGAGAAGCTGTACCTGTACTACCTGCAGAATGGGCGGGA
TATGTACGTGGACCAGGAACTGGACATCAACCGGCTGTCCGACTACGATGT
GGACCATATCGTGCCTCAGAGCTTTCTGAAGGACGACTCCATCGACAACAA
GGTGCTGACCAGAAGCGACAAGAACCGGGGCAAGAGCGACAACGTGCCC
TCCGAAGAGGTCGTGAAGAAGATGAAGAACTACTGGCGGCAGCTGCTGAA
CGCCAAGCTGATTACCCAGAGAAAGTTCGACAATCTGACCAAGGCCGAGA
GAGGCGGCCTGAGCGAACTGGATAAGGCCGGCTTCATCAAGAGACAGCTG
GTGGAAACCCGGCAGATCACAAAGCACGTGGCACAGATCCTGGACTCCCG
GATGAACACTAAGTACGACGAGAATGACAAGCTGATCCGGGAAGTGAAAG
TGATCACCCTGAAGTCCAAGCTGGTGTCCGATTTCCGGAAGGATTTCCAGT
TTTACAAAGTGCGCGAGATCAACAACTACCACCACGCCCACGACGCCTACC
TGAACGCCGTCGTGGGAACCGCCCTGATCAAAAAGTACCCTAAGCTGGAA
AGCGAGTTCGTGTACGGCGACTACAAGGTGTACGACGTGCGGAAGATGAT
CGCCAAGAGCGAGCAGGAAATCGGCAAGGCTACCGCCAAGTACTTCTTCT
ACAGCAACATCATGAACTTTTTCAAGACCGAGATTACCCTGGCCAACGGCG
AGATCCGGAAGCGGCCTCTGATCGAGACAAACGGCGAAACCGGGGAGATC
GTGTGGGATAAGGGCCGGGATTTTGCCACCGTGCGGAAAGTGCTGAGCAT

GCCCCAAGTGAATATCGTGAAAAAGACCGAGGTGCAGACAGGCGGCTTCA
GCAAAGAGTCTATCCTGCCCAAGAGGAACAGCGATAAGCTGATCGCCAGA
AAGAAGGACTGGGACCCTAAGAAGTACGGCGGCTTCGACAGCCCCACCGT
GGCCTATTCTGTGCTGGTGGTGGCCAAAGTGGAAAAGGGCAAGTCCAAGA
AACTGAAGAGTGTGAAAGAGCTGCTGGGGATCACCATCATGGAAAGAAGC
AGCTTCGAGAAGAATCCCATCGACTTTCTGGAAGCCAAGGGCTACAAAGA
AGTGAAAAAGGACCTGATCATCAAGCTGCCTAAGTACTCCCTGTTCGAGCT
GGAAAACGGCCGGAAGAGAATGCTGGCCTCTGCCGGCGAACTGCAGAAG
GGAAACGAACTGGCCCTGCCCTCCAAATATGTGAACTTCCTGTACCTGGCC
AGCCACTATGAGAAGCTGAAGGGCTCCCCCGAGGATAATGAGCAGAAACA
GCTGTTTGTGGAACAGCACAAGCACTACCTGGACGAGATCATCGAGCAGA
TCAGCGAGTTCTCCAAGAGAGTGATCCTGGCCGACGCTAATCTGGACAAA
GTGCTGTCCGCCTACAACAAGCACCGGGATAAGCCCATCAGAGAGCAGGC
CGAGAATATCATCCACCTGTTTACCCTGACCAATCTGGGAGCCCCTGCCGC
CTTCAAGTACTTTGACACCACCATCGACCGGAAGAGGTACACCAGCACCA
AAGAGGTGCTGGACGCCACCCTGATCCACCAGAGCATCACCGGCCTGTAC
GAGACACGGATCGACCTGTCTCAGCTGGGAGGCGACAAAAGGCCGGCGG
CCACGAAAAAGGCCGGCCAGGCAAAAAAGAAAAAGTAACCCGGGGGTAC
CCTGCAGGTCGACTTAATTAAGGATATGGCAGCTTAATGTTCGTTTTTCTTAT
TTATATATTTATACCAATTGATTGTATTTATAACTGTAAAAATGTGTATGTTGT
GTGCATATTTTTTTTTGTGCATGCACATGCATGTAAATAGCTAAAATTATGAA
CATTTTATTTTTTGTTCAGAAAAAAAAAAACTTTACACACATAAAATGGCTAG
TATGAATAGCCATATTTTATATAAATTAAATCCTATGAATTTATGACCATATTA
AAAATTTAGATATTTATGGAACATAATATGTTTGAAACAATAAGACAAAATTA
TTATTATTATTATTATTTTTACTGTTATAATTATGTGTCTCCTTCAATGATTCAT
AAATAGTTGGACTTGATTTTTAAAATGTTTATAATATGATTAGCATAGTTAAAT
AAAAAAAGTTGAAAAATTAAAAAAAAACATATAAACACAAATGATGGTTTT
TCCTTCAATTTCGATATCATATAGCAATCGTATTAGCAGGTAAATGAGCAAG
GTTAATAAAAAAATGGTAAAAAAAAAAAATTGGGGAAAAATAAACAAGGT
GTCTTTATCTACACTTTGGCTTTACAATGTATAATTTATATGCATACAAAAAA
ATTTTGATAAAATTGTAAAAAGGTGAATAAAATAACATTGTAACAGTAGTAG
ATAGTAAAGGGAAGGTGTTGCTCAAATAGTGTCGAAACAAAACTGGCATA
AAGAAAAATTGATATTGAGCAGAGGATATGCGCATAATGGTATTTTGTTTGT
TTGTTTGTTTTCTCATTTTTTGAGACAGCTCAATTCTTTATGTCCACAACATC
ATCGGACTTTTCTTCTTCAGGGTAGGCGGCCGC
(The green sequence: GFP ORF; the pink sequence: thosea asigna virus 2A peptide)

sequence of pLN-GFP

AAGCTTGGGGGGATCCTCTAGAGTCGACCTGCAGGCATGCTATTTGATGAA
TTAACTACACTTAAAATAATACAATTATTATTAAATTTTTTTTTGATTTATTTAT
TAATTTTTAAACTTAATCATTTGTATTTGGGAGGAATTATATATATCTTTATAAT
TATTTTATTTTTTTTTTATTTTTTTATTTTTTTATTATTATTATTTTTTTTTATTTTT
TTTTTTTACTGTATCAAAGAAAAACCTTTAAAAAAAAAAATTATAATTTCCCC

ATCTTACTATATTTTTAATACATACGTTTTAAGGAATTAAATTAGACAAAAGC
TATATTATGCTTTACATATAATTAGAATTTATAAACGTTTGGTTATTAGATATTT
CATGTCTCAGTAAAGTCTTTCAATACATATGTAAAAAAATATATATGAATACA
CATAAGTTGTTAATATATTTTATATGCATAAATGTATAAATATATATATATATATA
TATATATGTATGTATGTATATGTGTGTATATGAAATTATTTCAATGTTTAATTTT
TTAAATTTTAATTTTTTTTTTTTTTTTTTTTTTTTTTATTATGTATATTGATCTTTATT
ATTTAAATATTACTTTTTTCGTTTTTTCTTCTTTTTATTATTTTTTTTTTTTTTTA
TATTTTATACAAATGGTAATTCAAATAAAAGGTATAAATTTATATTTAATTTTC
TTTTATGGATAAATAAAAGAAAAATATAAATATATAAAAATATAAAAATATATA
TATGTATATTGGGGTGATGATAAAATGAAAGATAATATATATATATATATATCTT
TATTTTTTTTTTTTTTGTAGACCCCATTGTGAGTACATAAATATATTATATAACT
CGACCTTAAGTTATTTGTATAGTTCATCCATGCCATGTGTAATCCCAGCAGCT
GTTACAAACTCAAGAAGGACCATGTGGTCTCTCTTTTCGTTGGGATCTTTC
GAAAGGGCAGATTGTGTGGACAGGTAATGGTTGTCTGGTAAAAGGACAGG
GCCATCGCCAATTGGAGTATTTTGTTGATAATGGTCTGCTAGTTGAACGCTT
CCATCTTCAATGTTGTGTCTAATTTTGAAGTTAACTTTGATTCCATTCTTTTG
TTTGTCTGCCATGATGTATACATTGTGTGAGTTATAGTTGTATTCCAATTTGT
GTCCAAGAATGTTTCCATCTTCTTTAAAATCAATACCTTTTAACTCGATTCTA
TTAACAAGGGTATCACCTTCAAACTTGACTTCAGCACGTGTCTTGTAGTTCC
CGTCATCTTTGAAAAATATAGTTCTTTCCTGTACATAACCTTCGGGCATGGC
ACTCTTGAAAAAGTCATGCTGTTTCATATGATCTGGGTATCTCGCAAAGCAT
TGAACACCATAACCGAAAGTAGTGACAAGTGTTGGCCATGGAACAGGTAG
TTTTCCAGTAGTGCAAATAAATTTAAGGGTAAGTTTTCCGTATGTTGCATCA
CCTTCACCCTCTCCACTGACAGAAAATTTGTGCCCATTAACATCACCATCTA
ATTCAACAAGAATTGGGACAACTCCAGTGAAAAGTTCTTCTCCTTTACTCA
TGCCGGACGGACCACCCATCCTAGGTGATATATTTCTATTAGGTATTTATTATT
ATAAAATATAAATCTTGAATGATAATAAATAAAATATTAGTTATTCCTTTTCTA
GTTTAAAATATACATATTATAAATATATATATATATATATATATTTTTATTGTGAC
AAGAATATATAATTATAAATTATATTATTTATTTTTGTATTTTTTTTTTTTTTTTTT
TTTTTTTTCTTTTTTTGTTTTATTTTTCTTTTTTTTTATAAATATTATTTTTTTCT
TTTATCATGCACATTGGAATAATACATTAATATATATATATATATATTATATTATACA
TATATTGAATAATGTTTATAAAAAATGCATAACTTATATGAATATAATTTTTTTT
AAATATGACAAAAAGAAAAAAAAAAAAAAACCAAAAAAAAATTAAAATTGA
AATGAAATATATAAATATATTATTTATATATATTATACATTGTTTAATACTACTAC
ATGTATATATATATATTATATATATATATATATATCAATTTTTTCAAAAATAAATTA
ATATAAAAAGAGGGGAAAAAAAAAAAAAAAAAAAAAAAAAAGATAATTAA
GTAAGCATTTAAAAATATATAAATTGATAATATATAAAATTAATCACATATAAA
CTAATATAATTTATAAAATAAGGAAAATAAAATATTACCATAAAATAAAAATA
AAAATAAAAAAAAAAAAAAAAAAACACCTTTTTTTATATATATTAATATATAAT
TATCTCTTAGAAAAAATATTGTATAATTATATATGTAATGATTTATATAAAAAA
ATAAAATTATACAAGTATATATTTTGTTTCTATAAATTGATATCTTAATTATTTA
TTATTAGAAATAGATATTTTTATAATAAACCAATAGATAAAATTTGTAGAGAA
AAAAAAATAAAAATAAAAATAAAAATAATATAATATATAATAAAATAAAATAA
TATTATATAAATATATTTTAATTTTTTTTACAAAATGGTTAACAAAGAAGAAG

CTCAGAGGCATGCAAGCTTCGATCCATATAATTATTAATAGGTACTTTTTTTT
TTTATATATGGGAATTTCCTTATAGGGCCCGCATGCTTAGCTAATTCGCTTGT
AAGAGGTACTCTCGTTTATGCAAAACTATTTGATATAGCATTTTAACAAGTA
CACATATATATATGTAATATATATACTATATATATCTATTGCATGTGTACTAAGCA
TGTGCATGGCATCCCCTTTTTCTCGTGTTTAAAACAGTTTGTATGATAAAATA
TAAAGGATTTGAAAAAGAGAAAAAAATATATGATCTCATCCTATATAGCGCC
ATAATTTTTATTTGGGTTGAATAAAATTTTCTACTAAATTTAGGTGTAAGTAA
AATAATGGAATATATATAAGTACAATAAAAAAGTGCATAAATTAAAAAATTTT
TATAATAAATATTTTTTTTAAAAAAGTCAATAATAATATTAAATATATATAACA
CAGGATTATATATGTTCACTACAATTTTTTATATTATAATATAAATTCTTTTCAA
TTTTCATTTTATTTTACATACACTTTCCTTTTTTGTCACTATATTTTAATATTCA
CATATTTAGTTTAAATACTGGCTATTTCTTTCTACATTTGCTAGTAACAATTGT
GTAGTGCTTATATATATACACACACCTAAAACTTACAAACCATGGTGATAAAT
GCATGCCAAGCCTTTGTCTCAAGAAGAATCCACCCTCATTGAAAGAGCAAC
GGCTACAATCAACAGCATCCCCATCTCTGAAGACTACAGCGTCGCCAGCGC
AGCTCTCTCTAGCGACGGCCGCATCTTCACTGGTGTCAATGTATATCATTTT
ACTGGGGGACCTTGTGCAGAACTCGTGGTGCTGGGCACTGCTGCTGCTGC
GGCAGCTGGCAACCTGACTTGTATCGTCGCGATCGGAAATGAGAACAGGG
GCATCTTGAGCCCCTGCGGACGGTGCCGACAGGTGCTTCTCGATCTGCATC
CTGGGATCAAAGCCATAGTGAAGGACAGTGATGGACAGCCGACGGCAGTT
GGGATTCGTGAATTGCTGCCCTCTGGTTATGTGTGGGAGGGCTAAGGTACC
CCATTAAATTTATTTAATAATAGATTAAAAATATTATAAAAATAAAAACATAA
ACACAGAAATTACAAAAAAAATACATATGAATTTTTTTTTTGTAATCTTCCTT
ATAAATATAGAATAATGAATCATATAAAACATATCATTTTTCATTTATTTACAT
TTAAAATTATTGTTTCAGTATCTTTAATTTATTATGTATATATAAAAATAACTTA
CAATTTTATTAATAAACAATATATGTTTATTAATTCATGTTTTGTAATTTATGGG
ATAGCGATTTTTTTTACTGTCTGTATTTTTCTTTTTTAATTATGTTTTAATTGTA
TTTTATTTTTATTATTGTTCTTTTTATAGTATTATTTTAAAACAAAATGTTTTTT
CTAAGAACTTATAATAATAATAAATATAAATTTTAATAAAAATTATATTTATCT
TTTACAATATGAACATAAAGTACAACATTAATATATAGCTTTTAATATTTTTAT
TCCTAATCATGTAAATCTTAAATTTTTCTTTTTAAACATATGTTAAATATTTAT
TTCTCATTATATATAAGGACATATTTATTAAACCGCAGAGAAATCTAGAGGTA
CCGAGCTCGAATTCCGGGTTTGTACCGTACACCACTGAGACCGCGGTGGTT
GACCAGACAAACCAGAATTCGGGTTTGTACCGTACACCACTGAGACCGCG
GTGGTTGACCAGACAAACCAGAATTCTATAGTGAGTCGTATTACAATTCACT
GGCCGTCGTTTTACAACGTCGTGACTGGGAAAACCCTGGCGTTACCCAACT
TAATCGCCTTGCAGCACATCCCCCTTTCGCCAGCTGGCGTAATAGCGAAGA
GGCCCGCACCGATCGCCCTTCCCAACAGTTGCGCAGCCTGAATGGCGAATG
GCGCCTGATGCGGTATTTTCTCCTTACGCATCTGTGCGGTATTTCACACCGC
ATATGGTGCACTCTCAGTACAATCTGCTCTGATGCCGCATAGTTAAGCCAGC
CCCGACACCCGCCAACACCCGCTGACGCGCCCTGACGGGCTTGTCTGCTC
CCGGCATCCGCTTACAGACAAGCTGTGACCGTCTCCGGGAGCTGCATGTGT
CAGAGGTTTTCACCGTCATCACCGAAACGCGCGAGACGAAAGGGCCTCGT
GATACGCCTATTTTTATAGGTTAATGTCATGATAATAATGGTTTCTTAGACGT

CAGGTGGCACTTTTCGGGGAAATGTGCGCGGAACCCCTATTTGTTTATTTTT
CTAAATACATTCAAATATGTATCCGCTCATGAGACAATAACCCTGATAAATGC
TTCAATAATATTGAAAAAGGAAGAGTATGAGTATTCAACATTTCCGTGTCGC
CCTTATTCCCTTTTTTGCGGCATTTTGCCTTCCTGTTTTTGCTCACCCAGAAA
CGCTGGTGAAAGTAAAAGATGCTGAAGATCAGTTGGGTGCACGAGTGGGT
TACATCGAACTGGATCTCAACAGCGGTAAGATCCTTGAGAGTTTTCGCCCC
GAAGAACGTTTTCCAATGATGAGCACTTTTAAAGTTCTGCTATGTGGCGCG
GTATTATCCCGTATTGACGCCGGGCAAGAGCAACTCGGTCGCCGCATACACT
ATTCTCAGAATGACTTGGTTGAGTACTCACCAGTCACAGAAAAGCATCTTA
CGGATGGCATGACAGTAAGAGAATTATGCAGTGCTGCCATAACCATGAGTG
ATAACACTGCGGCCAACTTACTTCTGACAACGATCGGAGGACCGAAGGAG
CTAACCGCTTTTTTGCACAACATGGGGGATCATGTAACTCGCCTTGATCGTT
GGGAACCGGAGCTGAATGAAGCCATACCAAACGACGAGCGTGACACCACG
ATGCCTGTAGCAATGCCAACAACGTTGCGCAAACTATTAACTGGCGAACTA
CTTACTCTAGCTTCCCGGCAACAATTAATAGACTGGATGGAGGCGGATAAA
GTTGCAGGACCACTTCTGCGCTCGGCCCTTCCGGCTGGCTGGTTTATTGCT
GATAAATCTGGAGCCGGTGAGCGTGGGTCTCGCGGTATCATTGCAGCACTG
GGGCCAGATGGTAAGCCCTCCCGTATCGTAGTTATCTACACGACGGGGAGT
CAGGCAACTATGGATGAACGAAATAGACAGATCGCTGAGATAGGTGCCTCA
CTGATTAAGCATTGGTAACTGTCAGACCAAGTTTACTCATATATACTTTAGAT
TGATTTAAAACTTCATTTTTAATTTAAAAGGATCTAGGTGAAGATCCTTTTTG
ATAATCTCATGACCAAAATCCCTTAACGTGAGTTTTCGTTCCACTGAGCGTC
AGACCCCGTAGAAAAGATCAAAGGATCTTCTTGAGATCCTTTTTTTCTGCG
CGTAATCTGCTGCTTGCAAACAAAAAAACCACCGCTACCAGCGGTGGTTTG
TTTGCCGGATCAAGAGCTACCAACTCTTTTTCCGAAGGTAACTGGCTTCAG
CAGAGCGCAGATACCAAATACTGTCCTTCTAGTGTAGCCGTAGTTAGGCCA
CCACTTCAAGAACTCTGTAGCACCGCCTACATACCTCGCTCTGCTAATCCTG
TTACCAGTGGCTGCTGCCAGTGGCGATAAGTCGTGTCTTACCGGGTTGGAC
TCAAGACGATAGTTACCGGATAAGGCGCAGCGGTCGGGCTGAACGGGGGG
TTCGTGCACACAGCCCAGCTTGGAGCGAACGACCTACACCGAACTGAGAT
ACCTACAGCGTGAGCTATGAGAAAGCGCCACGCTTCCCGAAGGGAGAAAG
GCGGACAGGTATCCGGTAAGCGGCAGGGTCGGAACAGGAGAGCGCACGA
GGGAGCTTCCAGGGGGAAACGCCTGGTATCTTTATAGTCCTGTCGGGTTTC
GCCACCTCTGACTTGAGCGTCGATTTTTGTGATGCTCGTCAGGGGGGCGGA
GCCTATCGAAAAACGCCAGCAACGCGGCCTTTTTACGGTTCCTGGCCTTTT
GCTGGCCTTTTGCTCACATGTTCTTTCCTGCGTTATCCCCTGATTCTGTGGAT
AACCGTATTACCGCCTTTGAGTGAGCTGATACCGCTCGCCGCAGCCGAACG
ACCGAGCGCAGCGAGTCAGTGAGCGAGGAAGCGGAAGAGCGCCCAATAC
GCAAACCGCCTCTCCCCGCGCGTTGGCCGATTCATTAATGCAGCTGGCACG
ACAGGTTTCCCGACTGGAAAGCGGGCAGTGAGCGCAACGCAATTAATGTG
AGTTAGCTCACTCATTAGGCACCCCAGGCTTTACACTTTATGCTTCCGGCTC
GTATGTTGTGTGGAATTGTGAGCGGATAACAATTTCACACAGGAAACAGCT
ATGACCATGATTACGCCAAGCTATTTAGGTGACACTATAGAATACTC
(The green sequence: GFP ORF)
